# Sex Differences in IL-33-Induced STAT6-Dependent Type 2 Airway Inflammation

**DOI:** 10.3389/fimmu.2019.00859

**Published:** 2019-05-01

**Authors:** Hedi Zhao, Vanessa Moarbes, Véronique Gaudreault, Jichuan Shan, Haya Aldossary, Louis Cyr, Elizabeth D. Fixman

**Affiliations:** ^1^Meakins-Christie Laboratories, Research Institute of the McGill University Health Centre, Montréal, QC, Canada; ^2^Research Institute of the McGill University Health Centre, Montréal, QC, Canada

**Keywords:** innate immunity, eosinophil activation, type 2 innate lymphoid cells, IL-33, sex difference, STAT6

## Abstract

Sex differences in asthma prevalence are well-documented but poorly understood. Murine models have contributed to our understanding of mechanisms that could regulate this sex disparity, though the majority of these studies have examined responses present after Th2 adaptive immunity is established. We have now investigated how sex influences acute activation of innate cell populations in the lung upon initial exposure to the model antigen, ovalbumin (OVA), in the presence of IL-33 (OVA+IL-33), to prime the lungs for type 2 immunity. We also examined how inflammatory responses induced by OVA+IL-33 were altered in mice lacking the STAT6 transcription factor, which is activated by IL-13, an effector cytokine of IL-33. Our data demonstrate that type 2 inflammation induced by OVA+IL-33 was more severe in female mice compared to males. Females exhibited greater cytokine and chemokine production, eosinophil influx and activation, macrophage polarization to the alternatively activated phenotype, and expansion of group 2 innate lymphoid cells (ILC2s). While increases in ILC2s and eosinophils were largely *independent* of STAT6 in both males and females, many other responses were STAT6-dependent only in female mice. Our findings indicate that a subset of type 2 inflammatory responses induced by OVA+IL-33 require STAT6 in both males and females and that enhanced type 2 inflammation in females, compared to males, is associated with greater IL-13 protein production. Our findings suggest blunted IL-13 production in males may protect against type 2 inflammation initiated by OVA+IL-33 delivery to the lung.

## Introduction

Allergic asthma is a type 2-biased chronic inflammatory disease of the airways, historically considered to be orchestrated by CD4^+^ Th2 cells driving maladaptive responses in innate immune cells and structural cells of the lung (1). Recent interest has expanded to better understand mechanisms by which innate cells promote allergic airways disease, with particular attention on group 2 innate lymphoid cells (ILC2s) and the cytokines that activate them: IL-33, TSLP, and IL-25 (2).

Both the incidence and severity of asthma and allergies have been increasing steadily over the last two to three decades, with at least 300 million asthma sufferers worldwide and asthma ranking as one of the costliest of all chronic diseases (3). Asthma is more prominent in males during childhood, but this trend changes around the time of puberty, when asthma incidence declines in males and rises in females (4). Often times, the disease is considerably more severe or difficult to treat in females (5). Evidence from animal models suggests that estrogens and androgens may participate in this apparent *switch* (6, 7). Many studies of allergic airways disease in mice have shown enhanced eosinophilic inflammatory responses in females (7–9). Consistent with the pro-inflammatory role of estrogens, eosinophilic inflammation in these models is reduced both by ovariectomy and treatment with tamoxifen, an estrogen antagonist (10). Most of these studies have relied upon systemic sensitization of mice with ovalbumin (OVA) in the presence of alum as a Th2-skewing adjuvant. However, more recently, enhanced airway inflammatory responses have been documented in female mice exposed to the clinically relevant allergen, house dust mite (HDM), delivered directly to the airways (without the use of adjuvant) (7, 9). Moreover, compelling new data have shown that androgens inhibit proliferation and cytokine production from ILC2s in the lung (7). Evidence that ILC2s do not participate in allergic airways disease in systemically sensitized mice (11) suggests that the contribution of androgens and ILC2s in sex-specific responses in murine models of asthma may have been overlooked.

ILC2s are innate lymphoid cells that lack antigen-specific receptors found on their CD4^+^ Th2 cell counterparts (12). Their presence in various organs—such as the lung, gut, or uterus—substantiates their importance in local immunity, tissue homeostasis, and metabolic regulation (13). It has been proposed that lung ILC2s can be divided into two subsets, inflammatory ILC2s (iILC2) and natural ILC2s (nILC2) based on expression of IL-25R (IL-17RB) and ST2, respectively (14). Despite the relative paucity of nILC2s in the lung mucosa, upon activation by IL-33 (alone or in conjunction with TSLP and/or IL-25), they proliferate extensively and produce large amounts of type 2 cytokines, most commonly IL-5 and IL-13 (15–17). These cytokines promote eosinophil influx into the lung and, through IL-13-dependent induction of dendritic cell migration to the lung draining lymph nodes, subsequent adaptive immunity in papain- or HDM-treated mice (18, 19). Consistent with these data, mice lacking ST2 or treated with a soluble ST2 receptor have diminished airway inflammatory responses in murine models of asthma (16, 19–21), likely due, at least in part, to loss of IL-33-induced responses in ILC2s.

Activation of lung ILC2s correlates with an increase in the number of eosinophils in the lung and greater recovery in the bronchoalveolar lavage fluid (BALF). This eosinophil recruitment is generally considered to be under the control of IL-5. Recently, Abdala-Valencia et al. demonstrated, using an OVA/alum model of allergic airways disease, that two distinct populations of CD45^+^Ly6G^−/int^SiglecF^+^ eosinophils that differentially express CD11c were present in the lungs of OVA-challenged mice (22). While two populations were present in the lung tissue, only eosinophils that were activated and expressed CD11c were recovered in the BALF (22). On the other hand, eosinophils lacking CD11c remained localized to the lung (22). These data reflect similar findings of Mesnil et al. who characterized eosinophil phenotype in mice exposed to HDM (23). Whether these phenotype changes occur in response to IL-33 and whether sex differences affect these responses prior to the onset of adaptive immunity does not appear to have been addressed.

The type 2 cytokine, IL-13, is considered the main effector cytokine in allergic asthma (24, 25). IL-13 also mediates many airway inflammatory responses induced by IL-33 (26, 27). Upon binding to its cognate receptor comprised of the IL-4 receptor alpha subunit (IL-4Rα) and the IL-13Rα1 subunit (expressed by both immune and structural cells in the lung), IL-13 initiates STAT6 signaling (28, 29). Once activated, STAT6 forms homodimers that translocate into the nucleus to promote gene expression, ultimately leading to chemokine production, eosinophil recruitment, goblet cell differentiation, and airway hyperresponsiveness (26, 30, 31). Macrophages in the lung respond to IL-13 by differentiating into alternatively activated macrophages (AAM), which are characterized by STAT6-dependent expression of arginase 1 (Arg1) and mannose receptor (CD206); and production of CCL17, CCL22, and CCL24 to recruit Th2 cells and eosinophils into the lung (26, 32). Enhanced AAM differentiation in females has been detailed in a number of studies (8, 33, 34). While most of these studies have assessed responses in humans or in mice in the presence of Th2 adaptive immunity, recent evidence suggests that enhanced AAM differentiation in females may be cell intrinsic, mediated by epigenetic mechanisms (34). Aside from the well-accepted role of STAT6 in inducing production of eosinophil (and Th2) chemokines from epithelial and myeloid cells (31) to promote recruitment of eosinophils into the lung, whether or not cell-intrinsic STAT6 activation modulates eosinophil phenotype or activity is not clear. Moreover, while ILC2s express both STAT6 and IL-13 receptors themselves (35, 36), it is not clear if STAT6 activation modulates responses in these cells.

We have now assessed how STAT6 contributes to type 2 inflammation induced by acute OVA+IL-33 delivery to the lung. We have delineated responses in macrophages, eosinophils and ILC2s, and compared responses in both wild type (WT) and STAT6-KO male and female mice. Our data reveal that, in response to IL-33, female mice exhibit a greater degree of type 2 inflammation in the form of IL-13 production, BALF inflammatory cell influx, lung eosinophil influx and activation, ILC2 proliferation, and AAM differentiation. Our data show that aspects of IL-33-induced type 2 inflammation—in both male and female mice—require STAT6. Moreover, our data suggest that male mice are protected from type 2 inflammation induced upon OVA+IL-33 exposure, at least in part, through control of IL-13 protein production.

## Materials and Methods

### Mice

Female and male, wild type BALB/c mice and STAT6-KO mice on a BALB/c background (originally from The Jackson Laboratory, Bar Harbor, ME), were bred in-house at the RI-MUHC. Mice were kept in pathogen-free conditions in cages with irradiated food and water. Animal studies were approved by the McGill University Animal Care Committee and performed following guidelines of the Canadian Council on Animal Care. At the time of treatment, all mice were 6–8 weeks old. In conducting research using animals, the investigators adhere to the laws of the United States and regulations of the Department of Agriculture.

### IL-33-Induced Airway Inflammation

Mice were treated intranasally, following brief isoflurane anesthesia, daily for each of 2 days with OVA (50 μg), either alone (as the negative control) or with IL-33 (0.5 μg) in a volume of 30 μl. IL-33 was purchased from Thermo Fisher Scientific (Carlsbad, CA) and OVA was purchased from Worthington Biochemical Corp (Lakewood, NJ). Mice were allowed to rest for 96 h following the last treatment, after which they were sacrificed by a lethal dose of sodium pentobarbital.

### Airway Hyperresponsiveness (AHR) and Histology

To assess lung function, mice were anesthetized using xylazine and sodium pentobarbital and paralyzed with pancuronium bromide. Total lung elastance and resistance in response to increasing doses of methacholine was measured as previously described (37, 38). Briefly, anesthetized and paralyzed mice were attached to a computer-controlled small-animal ventilator (flexiVent; Scireq, Montréal, QC, Canada). Baseline respiratory system resistance and elastance and maximal resistance and elastance to increasing doses of nebulized methacholine were recorded. For histology, lungs were perfused with PBS and inflated with 10% formalin through the trachea, after which the entire lungs were isolated and placed in formalin. Following embedding in paraffin, 0.5 μm sections were cut and mounted on glass slides. Slides were stained with haematoxylin and eosin (H&E) or periodic acid-Schiff (PAS) and examined under a microscope (Olympus BX 51).

### BALF Acquisition and Cell Counting

Following euthanasia, a vertical incision was made at the neck to access the trachea. The trachea was then cannulated with a 20-gauge metal catheter and fastened with surgical thread to prevent backflow. BALF was collected by connecting a 1 mL syringe to the catheter and lavaging the lungs two times with 1 mL of ice-cold PBS. From the first lavage, the supernatant containing the cytokines was separated from the cells by centrifugation at 500 RCF for 5 min. The supernatant from the second lavage was discarded and the remaining cells were combined with the cells from the first lavage. The total number of live and dead cells recovered was quantified using a hemocytometer and trypan blue staining, after which inflammatory cell populations were identified following cell spinning (Cytospin^TM^) and Diff-Quik staining for differential cell counting; this method was used to quantify the relative proportion of macrophages, eosinophils, neutrophils, and lymphocytes in the BALF based on morphologic criteria. The total cell count was multiplied by the proportion of each cell type to quantify the absolute number of each cell type in the BALF.

### Lung Digestion

Lungs were allocated for flow cytometry and lung explant culture in RPMI-1640 media with 10% FBS. Lung tissue was dissociated and enzymatically digested with a cocktail of DNase I (200 μg/ml; Sigma-Aldrich, Oakville, ON), Liberase^TM^ (100 μg/ml; Roche, Indianapolis, IN), hyaluronidase 1a (1 mg/ml; Life Technologies, Carlsbad, CA), and collagenase XI (250 μg/ml; Life Technologies) for 30 min at 37°C and 5% CO_2_ (39, 40). Afterward, cells were washed with RPMI-1640 media containing 1% Penicillin/Streptomycin and 5% fetal bovine serum (FBS) to maintain cell viability. Red blood cells were lysed with sterile, filtered ammonium-chloride-potassium (ACK) buffer, after which the remaining viable cells were recovered following filtration through a 0.7 μM strainer.

### Lung Explant Culture

The lung explant culture was prepared by allotting 2 million viable cells to each of two conditions, saline or IL-33. Cells were incubated for 48 h in a total of 1 mL of RPMI-1640 media with 10% FBS, 5% penicillin/streptomycin, 1 mM sodium pyruvate, 1 mM non-essential amino acids, and 55 μM 2-mercaptoethanol, containing either control saline or IL-33 (20 ng/ml) at 37°C and 5% CO_2_. The supernatants were collected and IL-13 and IL-5 production were quantified by ELISA.

### ELISA—Enzyme-Linked Immunosorbent Assay

IL-13 and IL-5 were quantified following the Ready-SET-Go! IL-13 and IL-5 ELISA kit instructions from Thermo Fisher Scientific (Carlsbad, CA). In duplicates, samples from OVA-treated mice were prepared neat, whereas samples from OVA+IL-33 treated mice were prepared with dilutions of 1:5 to 1:20 to ensure they fell within the detection limits of the assay: 3.5 to 500 pg/ml The levels of cytokines (including IL-13 and IL-5) and chemokines in BAL fluid were assayed by mouse custom Q-Plex array (Quansys Biosciences, Logan UT). The plates were read with Q-View Imager LS and analyzed with Q-View Software (Quansys Bioscience). Samples below the limit of detection were assigned a value of 50% of the limit of detection.

### qPCR—Real-Time Quantitative PCR

The qPCR assay was conducted in accordance to the Minimum Information for Publication of Quantitative Real-Time PCR Experiments (MIQE) guidelines (41). The inferior lobe of the right lung was allocated for qPCR. To preserve RNA integrity, immediately after dissection, lung tissue was flash frozen in liquid nitrogen and stored at −80°C. Total RNA was isolated with the phenol-chloroform method using TRIzol Reagent (Invitrogen, Carlsbad, CA) according to the manufacturer's instructions. RNA purity and concentration were assessed by spectrophotometry using NanoDrop 2000. All samples had absorbance ratio ≥2.0 for 260/280 and 260/230 nm. RNA integrity was visualized on a 1% agarose gel containing 0.12% (v/v) NaClO and 0.5 μg/ml ethidium bromide, following Aranda et al. (42). To remove genomic DNA, 1 μg of RNA was treated with dsDNAse from Thermo Scientific after which cDNA was generated with the Thermo Scientific Maxima cDNA synthesis kit (#M1669) using random hexamer primers in a 20 μL reaction volume. qPCR efficiency was assessed using pre-validated β*-actin* primer pair and a serial dilution of the templates. We chose an appropriate dilution in which amplification efficiency was 100 ± 10%. qPCR reactions used 4 μL of cDNA (pre-diluted 1:20), 5 μL of PowerUp SYBR Green Master Mix (Applied Biosystems #A25780, Carlsbad, CA) and 0.5 μL of each primer pair at 5 μM. All samples were amplified in duplicate with the StepOnePlus Real-Time PCR system from Applied Biosystems using a standard cycling mode with the anneal/extend cycle temperature set at 60°C. Purity of each set of primers was validated with a “no-template” control for every experiment. Each set of primers had been previously tested for the optimal annealing temperature and products electrophoresed on a 2% agarose gel to ensure expected amplicon size. Murine primer sequences for the genes of interest can be found in Supplemental Table 1. Levels of *IL-13, Arg1, Fizz1*, and *Ym1* mRNA, all of which encode markers of AAM differentiation, were calculated with the ΔΔCT method (43), normalized to the reference gene β*-actin*, and presented relative to levels in OVA-treated control mice.

### Multi-Color Flow Cytometry

Viable lung cells were enumerated using trypan blue exclusion and samples diluted to obtain 1.25 × 10^6^ cells for ILC2 staining and 1 × 10^6^ cells for macrophage and eosinophil staining. Cells were incubated in the dark for 20 min with eFluor780 viability dye (eBioscience), then incubated at 4°C for 10 min with anti-CD32/16 to block Fc receptors. ILC2s were stained using EF-450-Thy1.2, PECy7-CD127, PerCP-eF710-ST2, KLRG1-APC, and a combination of PE-conjugated antibodies to CD3, CD11c, CD11b, CD49b, CD45R, TCRyD, and Ly6G. Macrophages and eosinophils were stained together with the following cocktail: BUV395-CD45.2, Alexa Fluor 700-Ly6G, BV510-F4/80, eFlour 450-MHCII, FITC-CD38, PeCy5.5-CD11c, PeC7-CD11b, APC-EGR2, PE-Siglec F, and PerCP-710 ST2. Additional information about the antibodies can be found in Supplemental Table 2. Cells were then fixed with IC fixation buffer (eBioscience) overnight before acquisition with BD LSRFortessa^TM^ (Immununophenotyping core facility, RI-MUHC) flow cytometer. Analysis was completed with FlowJo V10 (FlowJo LLC, Ashland OR). Fluorescence minus one (FMO) controls were used to define positive populations.

### Statistical Analysis

Graphs were generated using Graphpad Software and data were analyzed using two-way ANOVA. Multiple comparisons were performed using Tukey's *post hoc* test. A *p* ≤ 0.05 was considered significant. Outliers were removed using Grubb's test with an alpha of 0.05.

## Results

### Lung Delivery of IL-33 Increases BALF Eosinophil Influx in a Sex- and STAT6-Dependent Manner

**Figure 1:**
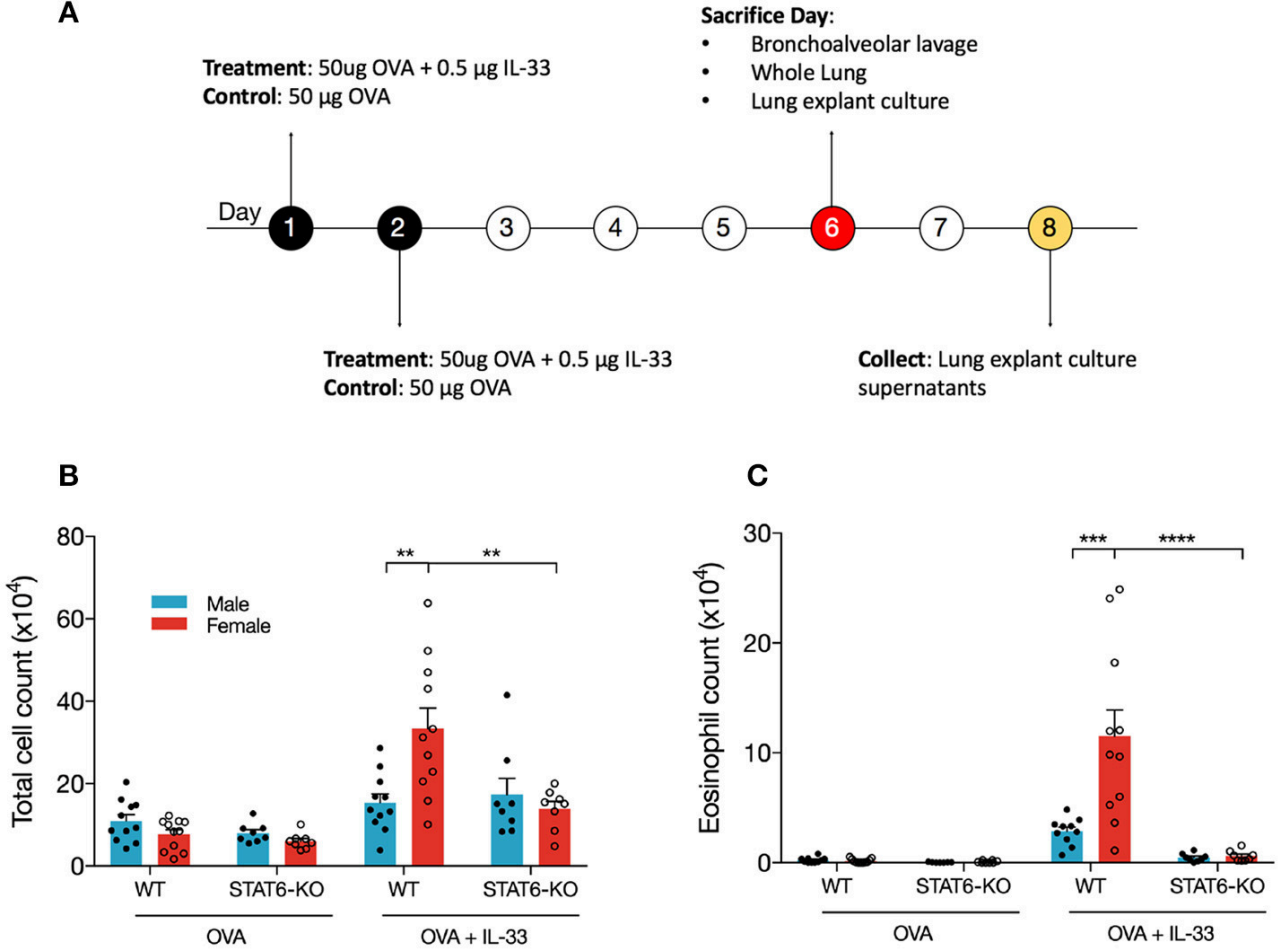
IL-33 induces airway inflammation and eosinophilia in females in a STAT6-dependent manner. **(A)** BALB/c mice (WT and STAT6-KO) were treated twice daily with OVA or OVA+IL-33 and sacrificed 96 h after the last treatment. **(B)** Absolute number of BALF cells was determined as described in the materials and methods section. **(C)** BALF differential count of eosinophils. Data are the combination of five independent experiments with 8–11 mice per group. Data are presented as mean + SEM. Two-way ANOVA, Tukey's *post hoc* test. **p ≤ 0.01, ***p ≤ 0.001, ****p ≤ 0.0001.

IL-33, which is released primarily from the epithelial cells in the lung in response to danger signals (e.g., allergens, viral infection), induces the release of type 2 cytokines, including IL-13, from several innate immune cells in the lung that then promote lung inflammation (44). To better understand how STAT6 and sex influence type 2 inflammation initiated by IL-33, we treated male and female mice on WT and STAT6-KO backgrounds with either OVA as a control, or OVA+IL-33 according to the timeline shown in Figure 1A. OVA was included with IL-33 to more closely mimic the *in vivo* response to danger signals in which IL-33 is naturally released in the presence of antigen. First, the profile of inflammatory cells in the BALF of mice treated with OVA or OVA+IL-33 was assessed. As expected, in mice treated with OVA alone, there were no significant differences between males and females or between WT and STAT6-KO mice: very few eosinophils were present and the majority of BALF cells were macrophages (Figures 1A,B and data not shown). In mice treated with OVA+IL-33, WT females had significantly more total BALF cells (Figure 1B) *and* eosinophils (Figure 1C) compared to WT males. Total BALF cells and eosinophils were significantly reduced in the absence of STAT6 in female mice (Figures 1B,C); in males, there was a clear trend for BALF eosinophils to be reduced, though this difference did not reach statistical significance (Figure 1C).

BALF inflammation was similar in mice treated with IL-33 alone (Supplemental Figures 1A,B).

### Activation of Eosinophils in the Lung Is Regulated by STAT6 and Sex

**Figure 2:**
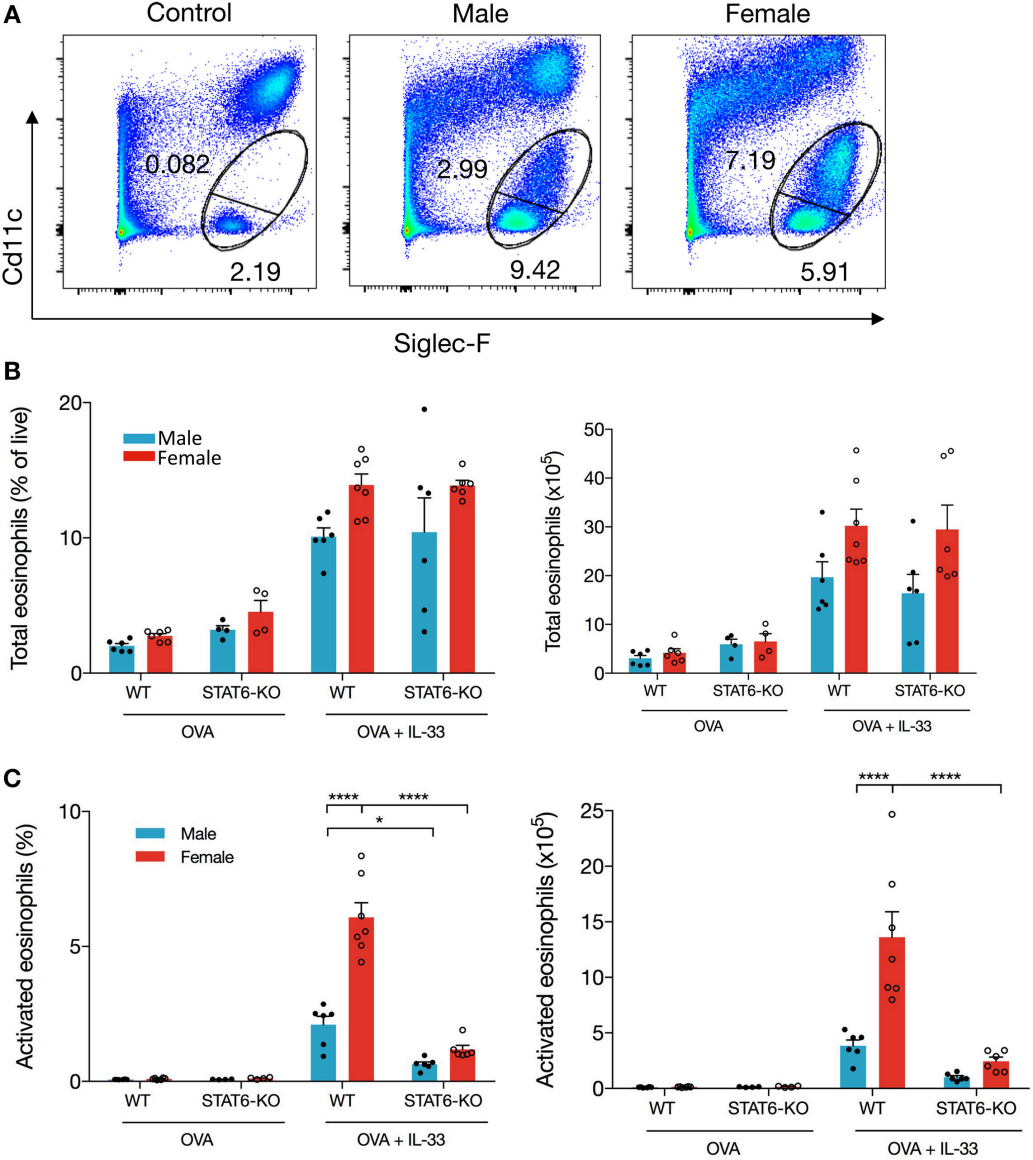
IL-33 induced eosinophil activation is dependent on STAT6. Mice were treated as in Figure 1A. Total lung cells were stained for eosinophils. **(A)** Representative dot plots of each treatment condition. **(B)** Total eosinophils represented both as a percentage of live cells (left panel) and total number (right panel). **(C)** Activated eosinophils represented as percentage of live cells (left panel) and total number (right panel). Data are from a combination of two experiments with 6–7 mice per group (except for OVA-treated STAT6-KO mice where *n* = 4 per group). Data are presented as mean + SEM. Two-way ANOVA, Tukey's *post hoc* test. *p ≤ 0.05, **p ≤ 0.01, ****p ≤ 0.0001.

To investigate the mechanism of differential eosinophil recruitment into the BALF of male and female mice and why these cells were almost completely absent in STAT6-KO mice, we examined the phenotype of eosinophils localized to the lung. Previous data have shown that eosinophils upregulate CD11c upon activation (22, 23) and that only these activated cells transit into the lumen of the airways where they can be recovered in the BALF (22). The proportion of total eosinophils, defined by the combination of CD11c-negative “mature” eosinophils (22) and CD11c-low “activated” eosinophils (22) (Figure 2A), was not significantly different between any of the IL-33-treated mice (Figure 2B), suggesting that OVA+IL-33-induced recruitment of eosinophils to the lung is both sex- and STAT6-independent. Activation of eosinophils, however, was differentially regulated by sex: activated eosinophils comprised 5.4 ± 0.4% of lung cells in female mice compared to 2.6 ± 0.1% in the males (Figure 2C). Consistent with the near absence of eosinophils in the BALF (Figures 1B,C), STAT6-KO mice *of either sex* had significantly reduced eosinophil activation (Figure 2C). When the proportions were converted into absolute cell numbers, the findings were the same: larger numbers of activated eosinophils were present in the lungs of females, and these numbers were reduced in STAT6-KO mice of either sex (though this difference was not significant in males). Together, these data indicate that recruitment and/or expansion of eosinophils in both male and female mice was increased with OVA+IL-33 in a STAT6-independent manner and that subsequent activation was dependent upon STAT6 and was greater in females.

### Lung Delivery of IL-33 Increases BALF Type 2 Cytokines and Chemokines in a Sex- and STAT6-Dependent Manner

**Figure 3:**
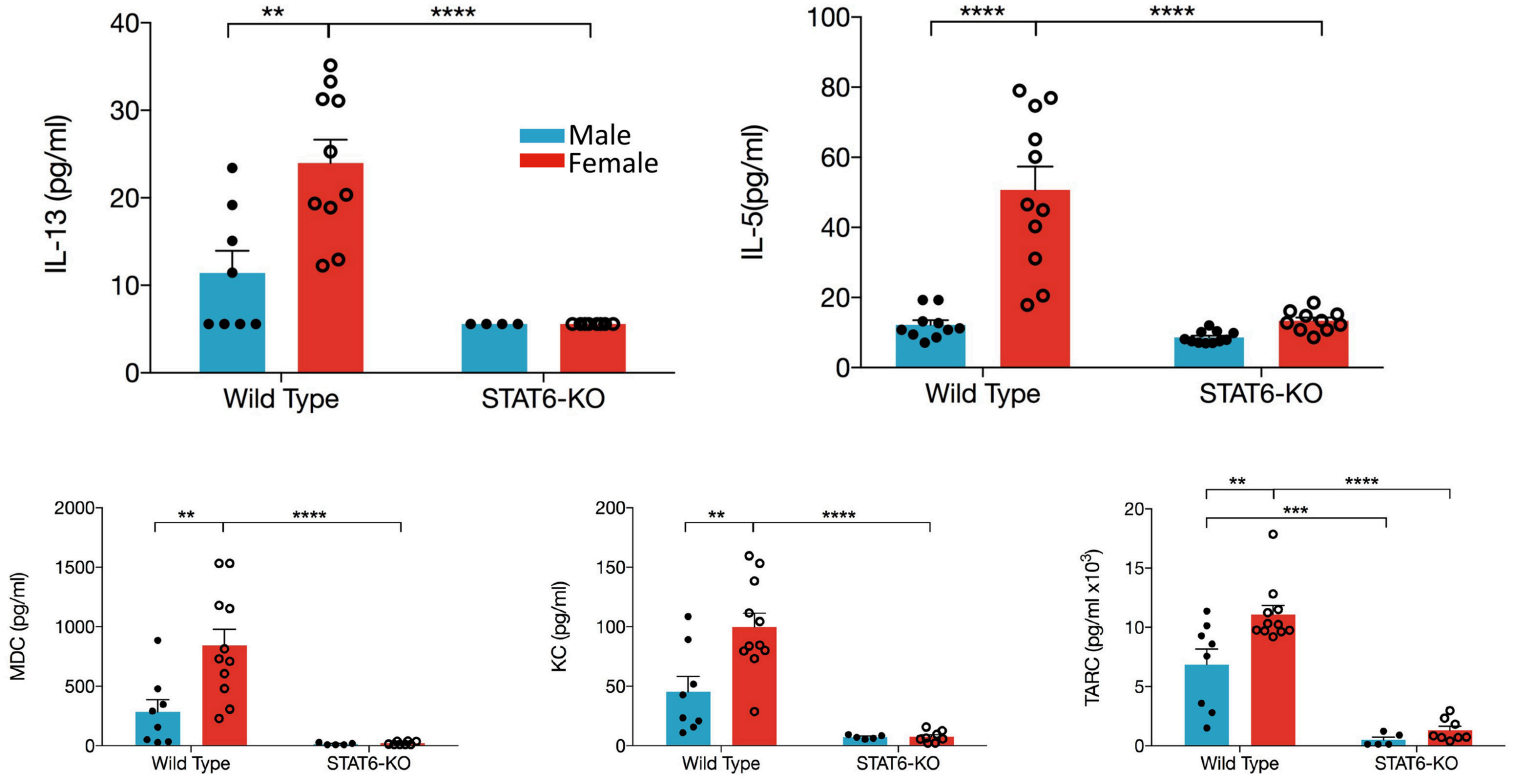
Type 2 cytokines and chemokines in the BALF are increased in female mice. Mice were treated as in Figure 1A. BALF was collected and IL-13, IL-5, CCL22/MDC, CXCL1/KC, and CCL17/TARC levels were quantified. Data are the combination of five independent experiments (*n* = 4–11) and presented as mean + SEM. Two-way ANOVA, Tukey's *post hoc* test. **p ≤ 0.01, ***p ≤ 0.001, ****p ≤ 0.0001.

IL-33 induces production of type 2 cytokines and chemokines, which then induce recruitment and/or activation of inflammatory cells from both the innate and adaptive arms of immunity. Our data demonstrate that, in WT females, significantly greater quantities of IL-13, IL-5, CCL17/TARC, and CCL22/MDC were present in the BALF compared to males (Figure 3). The neutrophil chemokine, CXCL1/KC was also significantly elevated in female mice, consistent with elevations of BALF neutrophils in female mice only (data not shown). Moreover, only in female mice were levels of IL-5, IL-13, CXCL1/KC, and CCL22/MDC significantly reduced by the absence of STAT6; only the Th2 chemokine (CCL17/TARC) exhibited STAT6 dependency in males (Figure 3). IL-4, IFNγ, IL-17, as well as CCL11 were undetectable in the BALF of all mice.

### IL-13 and IL-5 Protein Are Greatly Increased in Lung Cells From Female Mice Cultured *ex vivo*

**Figure 4:**
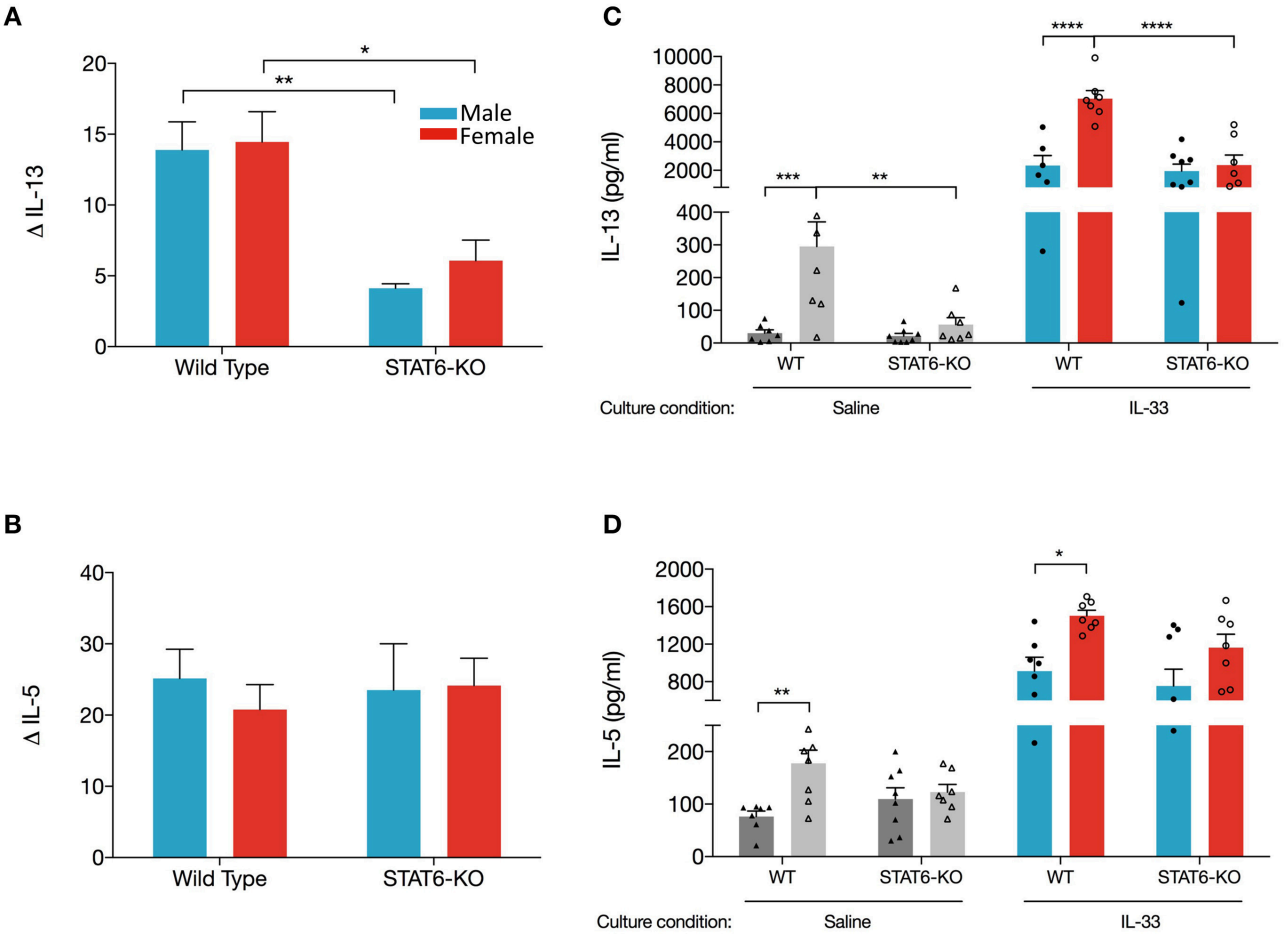
Lungs cell from female mice have greater ability to produce IL-13 when re-stimulated with IL-33 *ex vivo*. Mice were treated as in Figure 1A. Total lung RNA was isolated and qPCR performed. Levels of **(A)** *IL-13* and **(B)** *IL-5* mRNA were calculated with the ΔΔCT method, normalized to β*-actin*, and presented relative to levels in OVA-treated control mice. **(C,D)**. Total lung cells were cultured with either saline or IL-33 for 48 h and IL-13 and IL-5 were quantified from supernatants. Gray and colored bars represent cells cultured in saline and IL-33, respectively. Data are the combination of two independent experiments with 5–8 mice per group. Data are presented as mean + SEM. Two-way ANOVA, Tukey's *post hoc* test. *p ≤ 0.05, **p ≤ 0.01, ***p ≤ 0.001, ****p ≤ 0.0001.

Data presented in Figures 1, 2 revealed that sex-specific (and STAT6-dependent) differences in eosinophils in OVA+IL-33-treated mice were dependent upon which lung compartment was sampled (lung tissue vs. BALF). We also considered the possibility that the sex- and STAT6-specific differences in IL-13 and IL-5 in the BALF might not reflect levels in the lung. To address this question, we first quantified levels in the lung of mRNA encoding each of these cytokines. Firstly, our data showed that IL-13 mRNA levels were increased to a similar extent (~15-fold) in males and females treated with OVA+IL-33; this induction was significantly reduced in STAT6-KO mice of either sex (Figure 4A), suggesting that IL-13 mRNA levels may be promoted by a STAT6-dependent positive feedback loop. Interestingly, IL-5 mRNA levels were increased to the same extent by OVA+IL-33 in both males and females (~20-fold), but unlike IL-13, levels of IL-5 mRNA did not exhibit STAT6-dependence (Figure 4B).

Next, we examined production of IL-13 and IL-5 from total lung cells harvested from mice treated *in vivo* with OVA+IL-33 and cultured *ex vivo* with either saline or IL-33. We reasoned that cytokine production from lung cells cultured with saline would reflect *in vivo* levels produced by lung cells activated and/or recruited by *in vivo* delivery of OVA+IL-33. *Ex vivo re-stimulation* with IL-33 would reflect the potential of these cells to respond to IL-33 without the possibility of further recruitment of new cells to the lung. Our data show that IL-33-stimulated cultures from WT female mice produced the greatest quantity of IL-13 (7,036 ± 565 pg/ml), three times more than cultures from WT males (2,343 ± 702 pg/ml) (Figure 4C). IL-33-stimulated cultures from STAT6-KO mice of either sex also produced abundant IL-13, the level of which was significantly lower only in females. IL-33-induced IL-5 was also greater in WT females compared to males, though these levels were not significantly reduced in STAT6-KO mice of either sex (Figure 4D). Similar patterns of IL-13 and IL-5 production were present in saline-stimulated cultures, though the levels were much lower, particularly for IL-13 (Figures 4C,D).

Altogether, these data suggest that lower levels of IL-13 protein in males are due to sex-specific differences in the processing of IL-13 mRNA in WT mice. Specifically, our data indicate that, in the absence of STAT6, IL-13 mRNA levels were reduced in response to OVA+IL-33 in a manner that was similar in both males and females. In females, IL-13 protein levels followed the same trend: they were increased by *in vivo* delivery of OVA+IL-33 and reduced in STAT6-KO mice. In the case of males, greater levels of IL-13 mRNA in WT mice did not lead to similar increases in IL-13 protein. In fact, our data suggest that IL-13 protein levels in males treated with OVA+IL-33 were *independent* of STAT6. Similar regulation did not occur for IL-5. While levels of both IL-5 mRNA and protein were increased by OVA+IL-33, the absence of STAT6 had little impact in mice of either sex.

### Alternatively Activated Macrophage Differentiation Induced by IL-33 Is STAT6-Dependent and Enhanced in Female Mice

**Figure 5:**
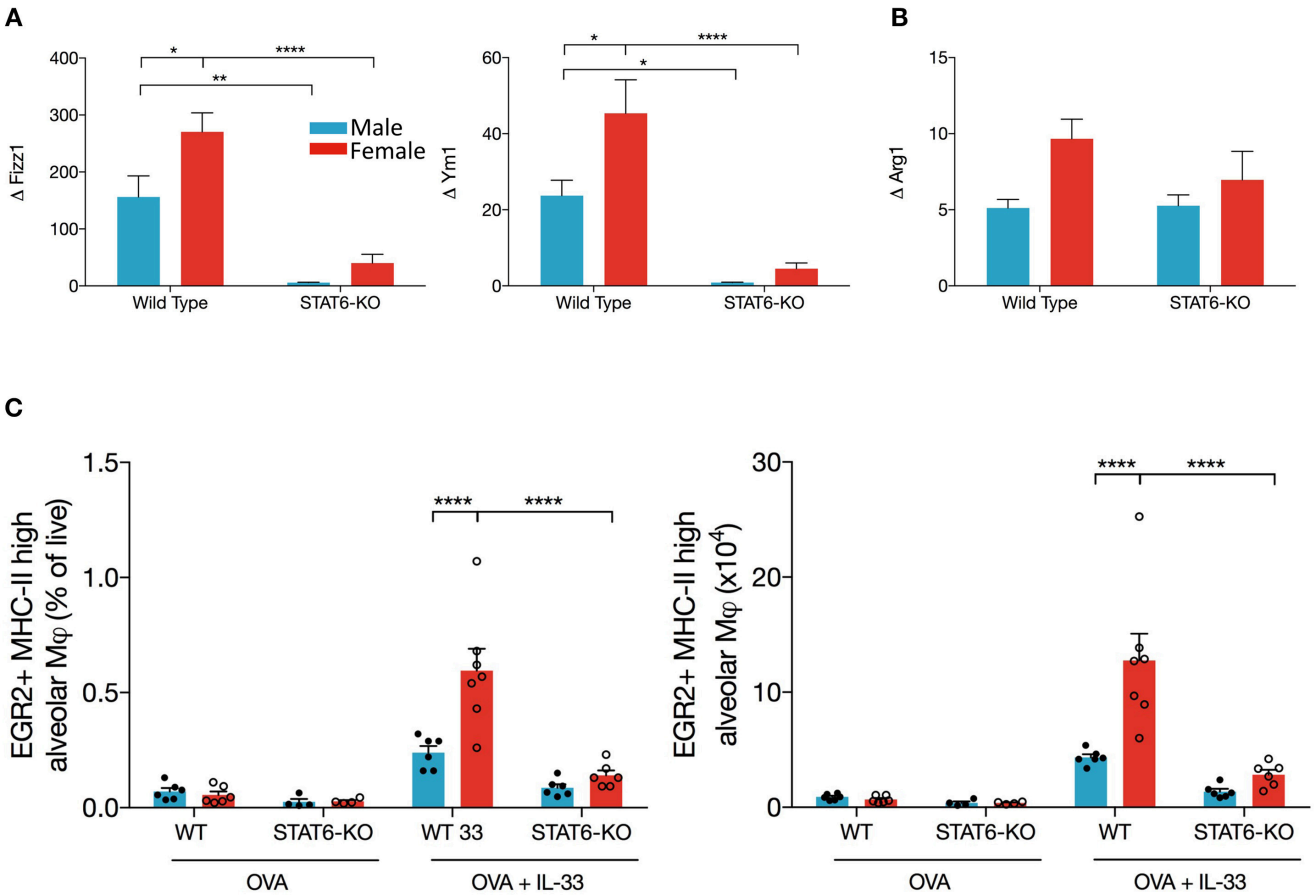
AAM differentiation is enhanced in female mice and is dependent upon STAT6. Mice were treated as in Figure 1A. Total lung RNA was isolated and qPCR performed. Levels of **(A)** *Ym1 and Fizz1* and **(B)** *Arg1* mRNA were calculated with the ΔΔCT method, normalized to β*-actin*, and presented relative to levels in OVA-treated control mice. **(C)** Total lung cells were stained for alveolar macrophages as described in the methods section. EGR2^+^MHCII^hi^ AAM are presented as a percentage of live (left panel) and absolute number (right panel). Data are from a combination of two experiments with 6–7 mice per group (except for OVA-treated STAT6-KO mice where *n* = 4 per group). Data are presented as mean + SEM. Two-way ANOVA, Tukey's *post hoc* test. *p ≤ 0.05, **p ≤ 0.01, ****p < 0.0001.

Our data suggest that IL-13 production in the lungs of OVA+IL-33-treated mice is partially dependent upon a STAT6 positive feedback loop. AAM are potential participants in this positive feedback loop: IL-33 induces polarization of alveolar macrophages to the AAM phenotype, which produce IL-13, via mechanisms that are at least partially dependent upon IL-13 and STAT6 (26, 45). Thus, we quantified mRNA levels of markers of AAM, *Arg1, Ym1*, and *Fizz1*, in OVA or OVA+IL-33 treated mice. *Ym1* and *Fizz1* mRNA levels were increased in both males and females, with levels significantly greater in females (Figure 5A) and each was dramatically reduced by the absence of STAT6 (Figure 5A). In contrast, *Arg1* mRNA levels, which tended to be greater in females, were not significantly affected by sex or STAT6 (Figure 5B), suggesting a non-AAM source of Arg1, such as ILC2s (35). The mRNA levels for these mediators were similarly regulated in mice treated with IL-33 alone (Supplemental Figure 1C). Flow cytometry analysis of AAM (gated as CD45^+^ Ly6G^int^ CD11c^hi^ SiglecF^+^ MHCII^hi^ Egr2^+^), revealed similar findings: greater AAM in females and reductions in STAT6-KO mice from either sex (Figure 5C), though total numbers of alveolar macrophages did not differ between mice of any group (data not shown). Thus, these data demonstrate that AAM differentiation in OVA+IL-33-treated mice is dependent upon both sex and STAT6, and provide evidence that these cells could contribute to IL-13 protein production in males and females exposed to IL-33 as well as the greater quantities of this cytokine in female mice.

### ILC2s Expand Dramatically in Female Mice Treated With IL-33, in a STAT6-Independent Manner

**Figure 6:**
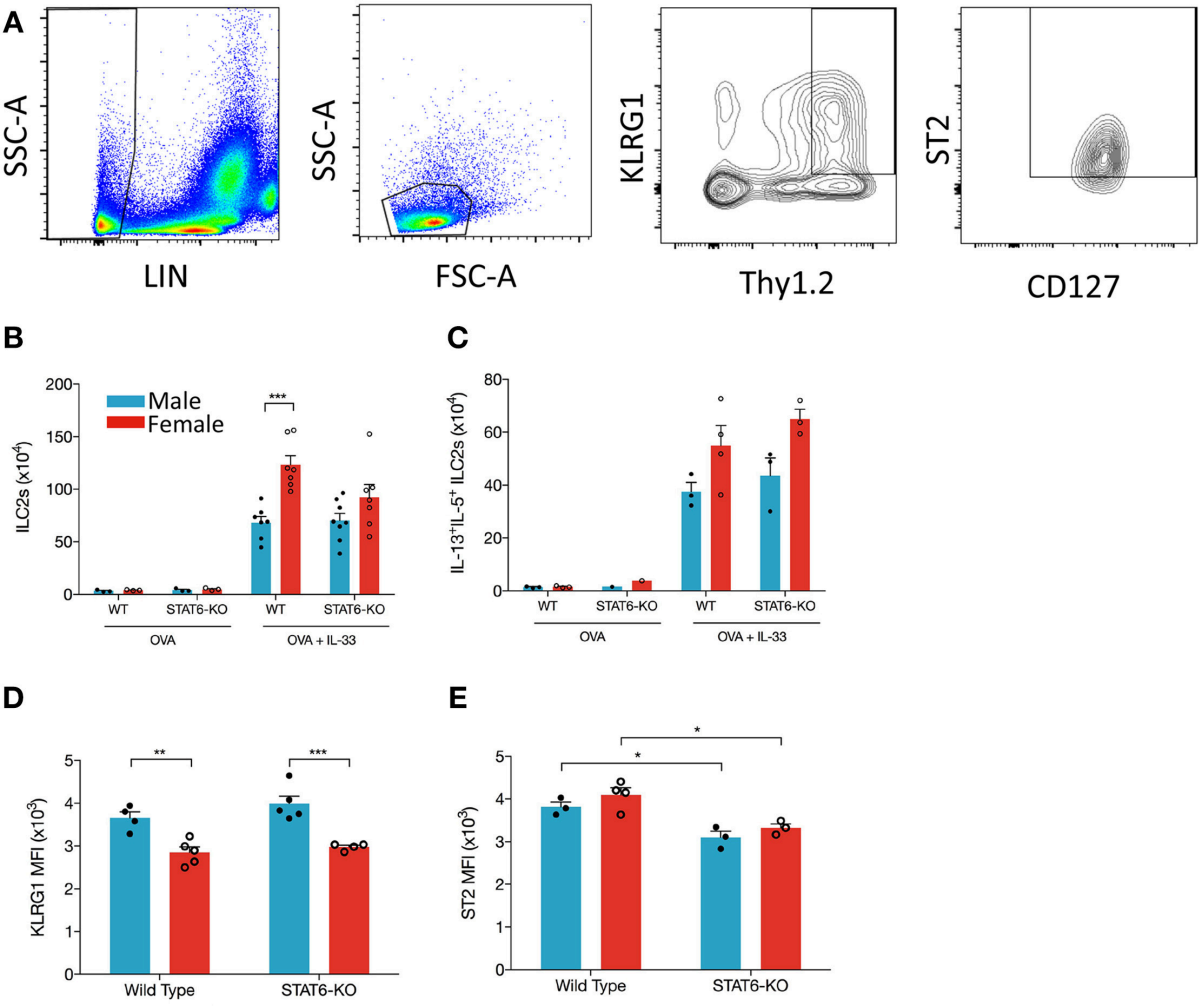
IL-33 induces ILC2 proliferation more effectively in female mice and is STAT6-independent. Mice were treated as in Figure 1A. Total lung cells were stained as described in the methods. **(A)** ILC2s were identified as Lin-, small lymphoid-like cells, Thy1.2^hi^KLRG1^+^ST2^+^CD127^+^ and quantified **(B)** as absolute cell number. **(C)** ILC2s expressing both IL-13 and IL-5 were quantified as absolute number. **(D)** KLRG1 MFI and **(E)** ST2 MFI of ILC2s were defined. Data in **(B)** are the combination of two independent experiments (*n* = 7–8), except for OVA controls (*n* = 3) and presented as mean + SEM. Date in **(C)** are from a single experiment (*n* = 3–4, expect STAT6-KO mice where *n* = 1). Data in **(D)** are from a single experiment (*n* = 4–5). Data in **(E)** are from a single experiment (*n* = 3–4). Two-way ANOVA, Tukey's *post hoc* test. *p ≤ 0.05, **p ≤ 0.01, ***p ≤ 0.001.

ILC2s are an obvious candidate to initiate each of the above responses through their production of IL-5 and IL-13, especially given recent data showing enhanced ILC2 responses in the lungs of female mice, both at baseline and following (intraperitoneal) IL-33 delivery (7). Whether the decreases in type 2 inflammation we identified in STAT6-KO mice were due to diminished responses in ILC2s themselves and/or “downstream” effector cells (e.g., eosinophils and AAM) was unclear. Thus, we examined whether intranasal OVA+IL-33 delivery differentially affected ILC2s in male and female mice and whether (the absence of) STAT6 diminished OVA+IL-33-dependent responses in these cells. We focused on so-called natural ILC2s expressing ST2 and high levels of Thy1.2 (following the gating strategy shown in Figure 6A). OVA-treated mice had negligible differences in ILC2s, whether they were from male or female, WT or STAT6-KO mice (Figure 6B). Upon intranasal delivery of OVA+IL-33 to WT mice, ILC2 numbers increased from ~3 × 10^4^ to ~68 × 10^4^ in males and ~4 × 10^4^ to ~123 × 10^4^ in females (Figure 6B). STAT6 played no role in either the expansion (Figure 6B) or potential for cytokine production (Figure 6C) of ILC2s in males. Although ILC2 numbers in OVA+IL-33 treated females tended to be lower in STAT6-KO mice (Figure 6B), this difference was no longer apparent when we quantified ILC2s expressing both IL-5 and IL-13 (Figure 6C). Similar to Laffont et al. (7), we found that ILC2s from males expressed higher levels of KLRG1 (Figure 6D), but STAT6 did not appear to play a significant role in this expression. The absence of STAT6 did lead to reductions in ST2 expression in both males and females (Figure 6E). Together, these data demonstrate that the losses of Type 2 inflammatory responses in STAT6-KO mice treated with OVA+IL-33 were not due to reduced ILC2 activation or cytokine production but instead due to losses of STAT6-dependent responses in ILC2 effector cells, such as eosinophils and AAM.

### IL-33 Induces Airway Hyperresponsiveness (AHR) in Both Males and Females and Enhanced Inflammation and Goblet Cell Hypertrophy/Metaplasia in Female Mice

**Figure 7:**
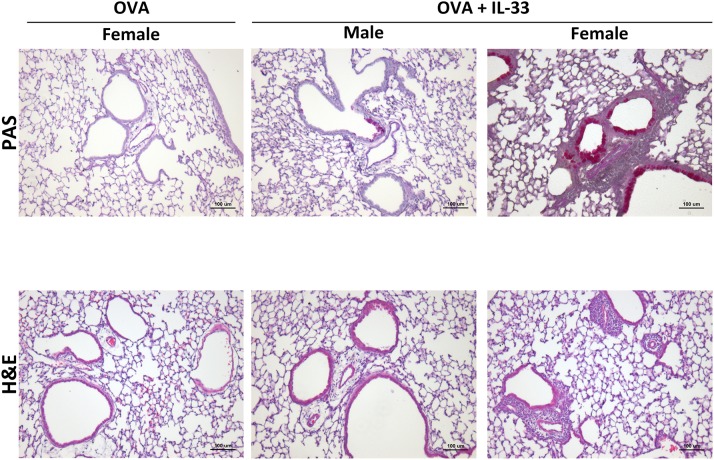
IL-33 induced inflammation and goblet cell hypertrophy/metaplasia are enhanced in female mice. Mice were treated with OVA or OVA+IL-33 as in Figure 1A. Top, PAS, and Bottom, H&E staining of lung airways. Magnification x10. Scale bar = 100 μm. Images shown are from one experiment and are representative of 3–4 mice per group. Male OVA-treated mice did not differ from similarly treated female mice.

To confirm enhanced responses in OVA+IL-33 treated female mice, AHR, inflammation and mucus levels were examined by invasive flexiVent technology and histology, respectively, in mice treated with OVA or OVA+IL-33. AHR was induced in both males and females (Supplemental Figure 2A). While there was a trend for both total lung elastance (Supplemental Figure 2A) and resistance (Supplemental Figure 2B) to be greater in females, this difference did not reach statistical significance. Finally, small amounts of inflammation and mucus production were apparent in males exposed to OVA+IL-33, consistent with the muted responses described above. However, in females, perivascular and peribronchial inflammation were prominent and goblet cell hypertrophy abundant after delivery of OVA+IL-33 to the lungs (Figure 7).

## Discussion

In this study, we have demonstrated that delivery of OVA+IL-33 to the lungs of mice induced an innate inflammatory response that exhibited sex-dependent differences in magnitude and regulation. Compared to males, female mice produced larger amounts of type 2 cytokines and chemokines and had substantially more activated eosinophils and AAM in the lung; each of these responses was significantly lower in female mice lacking STAT6. On the contrary, male mice displayed less inflammation overall, and in some instances, no apparent role for STAT6 was observed. BALF levels of inflammatory mediators and eosinophils were very low (and in many cases undetectable) in STAT6-KO mice, suggesting a near loss of responses in these mice. However, in analyzing the lung, comparable levels of eosinophils and ILC2s in both WT and STAT6-KO mice were found. These data provide evidence that STAT6 does not regulate OVA+IL-33-induced expansion of these inflammatory cell populations in the lung (through proliferation, *in situ* differentiation, and/or recruitment). Independent of the compartment sampled (BALF or lung) or outcome analyzed (e.g., cytokine levels, eosinophil activation, *ex vivo* cytokine production), female mice lacking STAT6 failed to sustain these sex-specific differences. While the magnitude of induction was consistently lower in male mice, many responses (e.g., *ex vivo* IL-13 production) were largely *independent* of STAT6 in these mice. Interestingly, male mice still exhibited robust AHR that was only marginally lower than that in female mice, supporting data from others who have found that lung inflammation in murine models can be uncoupled from AHR [e.g., (46)]. We speculate that ILC2s, which were also marginally lower in males likely participated in this response through their production of IL-13. Altogether, our data build upon our understanding of the canonical roles of STAT6 in type 2 inflammation in the lung (36).

The increase in lung inflammation was accompanied by robust cytokine production upon *ex vivo* IL-33 re-stimulation—even in STAT6-KO mice. *Ex-vivo* IL-13 protein production from lung cell cultures, whether exposed to saline or IL-33 was dependent in part on STAT6 in female, but not in male mice. In fact, we suggest that these findings are compatible with several possibilities: (1) female mice may have enhanced STAT6 activity that ultimately leads to heightened inflammatory responses in a number of innate cells; and/or (2) female mice may utilize alternative pathway(s) to promote STAT6-dependent IL-13 protein production, resulting in increased type 2 inflammation. In addition, males may possess pathways that selectively control IL-13 protein production, the outcome of which is less IL-13 and diminished type 2 inflammation. We did examine lung mRNA levels encoding several negative regulators of type 2 immunity. TGFβ and FoxP3 mRNA levels did not differ significantly under any condition (OVA vs. OVA+IL-33 or in wild-type vs. STAT6-KO mice—data not shown). Levels of IL-10 mRNA were increased in *females* treated with OVA+IL-33 and were greater in OVA+IL-33-treated females compared to their male counterparts (Supplemental Figure 3). Thus, it appears unlikely that any of these mediators is responsible for the sex differences observed in our model (acknowledging that protein levels were not quantified). Experiments are ongoing to define mechanisms by which IL-33-induced responses differ in males and females. Pathogenesis of allergic inflammation in males and females remains etiologically complicated; elucidating sex-specific differences that are present even before the onset of Th2 adaptive immunity will provide insight into drug development and targeted therapeutics that function more effectively and safely.

Abundant data from murine models demonstrate that clinically relevant allergens, through release of innate cytokines, including IL-33, activate ILC2s to proliferate and produce large quantities of IL-5 and IL-13 (7, 16, 18, 47, 48). Lung ILC2s promote dendritic cell migration to the lymph node, Th2 differentiation and cytokine production, eosinophil recruitment, mucus hypersecretion, IgE production, etc. each of which is reduced with a deficiency in the ILC2 compartment (48). We hypothesized that lung ILC2s initiated the sex differences observed in our model based on data from Laffont et al. who had shown that ILC2 numbers were lower in males (7). While they did not address whether STAT6 regulated ILC2s in this study, we considered that ILC2 cell-intrinsic STAT6 might be involved based on data from Doherty et al. who had shown that ILC2 (then called natural helper cells) responses were reduced in STAT6-KO mice in a model of Alternaria-induced lung inflammation (47). Our data confirmed larger numbers of ILC2s with trends for greater proportions with the potential to produce IL-5 and IL-13 in female mice treated with OVA+IL-33. Moreover, in STAT6-KO mice of either sex, ILC2 responses to OVA+IL-33 were equally robust compared to WT mice. Thus, we believe that the sex differences and the dependence upon STAT6 delineated in our study likely originate *downstream* of ILC2s. Eosinophils and AAMs are equipped with cognate receptors to respond to (ILC2-derived) IL-13 and, in the case of eosinophils, IL-5 (49, 50). Responses to IL-13 are enhanced by co-stimulation with IL-33 (26). Thus, we propose that IL-33 coordinates with IL-13 and possibly IL-5 to amplify IL-33-induced responses in each of these cells, ultimately leading to enhanced type 2 inflammatory responses in female mice.

Enhanced AAM differentiation in females is implicated in increased inflammation in murine models of allergic airways disease; there is also some evidence that these cells contribute to human disease (33, 34). To more clearly define a role for STAT6 in the sex differences we observed, we quantified mRNA levels for several widely used markers of AAM: *Arg1, Ym1*, and *Fizz1*. Our data showed that, while *Ym1* and *Fizz1* were dramatically upregulated in OVA+IL-33 treated mice, and to a greater extent in females, *Arg1* induction was much more modest. In addition, *Ym1* and *Fizz1* were almost completely STAT6-dependent in both males and females, whereas *Arg1* was not. This is likely due to the fact that ILC2s express *Arg1* constitutively, in a STAT6-independent manner (35). *Arg1* mRNA levels where more closely aligned with the data we obtained for ILC2s (numbers and cytokine production) in WT and STAT6-KO mice.

Our data are consistent with many reports showing that activated ILC2s participate in the recruitment of eosinophils to the lung, even in the absence of adaptive immunity (7, 16, 18, 48). IL-33 can act on eosinophils in the bone marrow directly or through production of IL-5 (51). Development, maturation, and migration of eosinophils from the bone marrow to the lung are most commonly regarded as outcomes of IL-5 signaling (51). ILC2 production of IL-13 could also promote eosinophil recruitment to the lung via STAT6-dependent production of chemokines, CCL11, and CCL24 (31). Thus, we hypothesized that the loss of eosinophils in the BALF of STAT6-KO mice was due at least in part to reduced production of STAT6-dependent chemokines and that fewer eosinophils would also be present in the lungs of these mice. To our surprise, we found that the total number of eosinophils in the lungs was both similar between males and females *and* completely independent of STAT6. Moreover, the lung eosinophil phenotype that most closely reflected the BALF eosinophil data was the upregulation of CD11c to the “activated” state proposed by Abdala-Valencia et al. (22), an increase that we show is almost completely STAT6 dependent. Thus, our data are consistent with a model in which IL-33 induces cytokine production from ILC2s, which leads to both recruitment of eosinophils to the lung (via IL-5) and their shift to an activated phenotype (via IL-13) promoting their migration into the air spaces. Aside from recruitment into the lung and ILC2 cytokine production, each of these responses is enhanced in females. There appears to be no role for STAT6-dependent chemokines in this early, innate recruitment of eosinophils into the BALF, consistent with our inability to detect CCL11.

It is well-established that STAT6 is a critical mediator of type 2 inflammation: Th2 differentiation and cytokine production, mucus hypersecretion, IgE class switching, AAM polarization, etc. Our experiments show that in the absence of STAT6, both IL-5 and IL-13 (as well as several chemokines) were dramatically reduced in the BALF; in re-stimulated lung cell cultures, IL-13 was also reduced in STAT6-KOs, though only in female mice. Medoff et al. showed that STAT6 in bone marrow derived myeloid CD11b^+^/CD11c^+^ cells was required for the production of chemokines that recruited Th2 cells and eosinophils to the lung (31). As discussed above, STAT6 has also been implicated in ILC2 proliferation as well as eosinophil recruitment to the BALF in a model of Alternaria-induced allergic inflammation (47). While we expected reductions in STAT6 KO mice, we were surprised to discover that several outcomes in male mice were only moderately, and often not significantly, reduced. Androgens have been linked to suppression of immune responses (7, 52, 53), and our data provide evidence that STAT6 could be linked to androgens in this sex difference. One possible mechanism could be the involvement of a phosphatase selectively regulated by androgen signaling. PTP1B (also known as PTPN1) is a protein tyrosine phosphatase, whose expression is amplified by androgens in prostate cancer cells and which dephosphorylates IGFR, Jak2, and Tyk2, kinases crucial to cytokine signaling pathways (54, 55). Despite the fact that these data originate from a distinct organ and disease, PTP1B expression is ubiquitous and selective modulation of its expression in endothelial cells has been implicated in allergic airways disease (56, 57). At high concentrations, it may interact with and dephosphorylate activated STAT6 (57, 58)—a scenario that, we propose, could be controlled by androgens, selectively in males. Likewise, Tyk2, which is activated by IL-13 receptors, is another putative target of PTP1B, which could also lead to dampening of IL-13-induced inflammatory responses.

Given the outcomes of IL-33-induced AAM differentiation, IL-13 production, and airway inflammation, the diversity of cells, transcription factors, and cytokines is complex, even without invoking adaptive immunity. While STAT6 modulates a number of the responses we observed, there are certainly other signaling pathways and transcription factors involved that we have not assessed. Our long-term goal is to define the inhibition of these (and other) IL-33-dependent responses using STAT6-IP, a cell penetrating peptide we developed to inhibit STAT6. Data from several studies have shown that STAT6-IP reduces Th2-biased inflammatory responses in the lung (38, 59, 60). Our published and unpublished data provide support for STAT6-IP inhibition of responses in innate cells of the lung to control Th2 adaptive immunity (37, 38, 60). Our ongoing objectives are to clarify how STAT6-IP modulates the ability of IL-33 to interact with IL-13 and STAT6 to induce AAM differentiation and eosinophil activation. This information will support further development of STAT6-IP for the treatment of allergic asthma in humans.

## Ethics Statement

Animal studies were approved by the McGill University Animal Care Committee and performed following guidelines of the Canadian Council on Animal Care.

## Author Contributions

HZ and EF were responsible for the design of the experiments and writing the manuscript. HZ, VM, VG, JS, HA and LC participated in data acquisition for the manuscript.

### Conflict of Interest Statement

The authors declare that the research was conducted in the absence of any commercial or financial relationships that could be construed as a potential conflict of interest.

## Abbreviations

AAM: alternatively activated macrophage(s)
BALF: bronchoalveolar lavage fluid
HDM: house dust mite
KO: knockout
nILC2: natural group 2 innate lymphoid cell
iILC2: inflammatory group 2 innate lymphoid cell
OVA: ovalbumin
STAT6: signal transducer and activator of transcription factor 6
WT: wild-type.
