# Supplementary material for: Sex Differences in IL-33-Induced STAT6-Dependent Type 2 Airway Inflammation
Source: Front Immunol. 2019 May 1;10:859. doi: 10.3389/fimmu.2019.00859 (PMC6504808; doi:10.3389/fimmu.2019.00859)
Supplement: Supplementary file 1 [file Data_Sheet_1.PDF]

Supplemental Table 1

qPCR Primers

Gene of Interest	Forward (5'-3')	Reverse (5'-3')	Company
Arg1	ACCTGGCCTTTGTTGATGTC	CAGCACCACACTGACTCTTC	IDT
Fizz1	CTGGGTTCTCCACCTCTTCAR	TGCTGGGATGACTGCTACTG	IDT
Ym1	CATCAGCAAGACTTGCGTGACR	GGTCCAAACTTCCATCCTCCA	IDT
IL-13	CCTGGCTCTTGCTTGCCTT	GGTCTTGTGTGATGTTGCTCA	IDT
iNOS	GTCTTTGACGCTCGGAACTGT	GATGGCCGACCTGATGTTG	IDT
B-actin	AGCCATGTACGTAGCCATCC	CTCTCAGCTGTGGTGGTGAA	IDT
IL-5	TCCAATGCATAGCTGGTGATTT	AGCACAGTGGTGAAAGAGACCTT	Invitrogen

Supplemental Table 2

Flow Cytometry Antibodies

Antibody	Conjugation	Clone	Company
CD11b	PE	M1/70	eBioscience
CD11c	PE	N418	BioLegend
CD3e	PE	145-2C11	BioLegend
CD49b	PE	DX5	BD Biosciences
CD45R	PE	RA3-6B2	Invitrogen
TCRyD	PE	GL3	BD Biosciences
Ly6G	PE	1A8	BioLegend
Thy1.2	EF450	53-2.1	Invitrogen
CD127	PECy7	A7R34	BioLegend
ST2	PerCP-EF710	RMST2-2	eBIoscience
KLRG1	APC	2F1/KLRG1	BioLegend
IL-5	APC		BD Biosciences
IL-13	AF488	eBio13A	eBioscience
