# Supplementary material for: Sex Differences in IL-33-Induced STAT6-Dependent Type 2 Airway Inflammation
Source: Front Immunol. 2019 May 1;10:859. doi: 10.3389/fimmu.2019.00859 (PMC6504808; doi:10.3389/fimmu.2019.00859)
Supplement: Supplementary file 2 [file Image_1.pdf]

HZhao et al
Sex differences in IL-33-induced STAT6-dependent type 2 airway inflammation

Supplemental Figure Legends

Supp Fig 1. IL-33 induces airway inflammation and eosinophilia in females in a STAT6-dependent manner
BALB/c mice (WT and STAT6-KO) were treated twice daily with IL-33 and sacrificed 96 hours after the last treatment. **A.** Absolute number of BALF cells and **B.** total BALF eosinophils were quantified. **C.** Total lung RNA was isolated and qPCR performed. Levels of *Ym1, Fizz1* and *Arg1* mRNA were calculated with the ΔΔCT method, normalized to *β-actin,* and presented relative to levels in OVA-treated control mice. Data are the combination of 4 independent experiments (a subset of which used only males or females) with a total of 5-8 mice per group. Data are presented as mean + SEM. Two-way ANOVA, Tukey's post hoc test. * ≤ 0.05, ** ≤ 0.01, *** $p \leq 0.001$, **** $p \leq 0.0001$.

Supp Fig 2. AHR is increased in both male and female mice treated with OVA+IL-33
Wild-type BALB/c mice were treated twice daily with OVA or OVA+IL-33 and sacrificed 96 hours after the last treatment. B. Lung function was assessed with the flexiVent small animal ventilator and is presented as **A.** lung elastance: dose response (left) and 25mg/ml dose (right) and **B.** lung resistance: does response (left) and 25mg/ml dose (right). Data are the combination of 2 independent experiments with a total of 4-7 mice per group. Data are presented as mean + SEM. One-way ANOVA, Tukey's post hoc test. * ≤ 0.05, ** ≤ 0.01, *** $p \leq 0.001$.

Supp Fig 3. IL-33 induces airway inflammation and eosinophilia in females in a STAT6-dependent manner
BALB/c mice (WT and STAT6-KO) were treated twice daily with OVA or OVA+IL-33 and sacrificed 96 hours after the last treatment. Total lung RNA was isolated and qPCR performed. Levels of IL-10 mRNA were calculated with the ΔΔCT method, normalized to *β-actin,* and presented relative to levels in OVA-treated control mice. Data are the combination of 2 independent experiments with a total of 3-8 mice per group. Data are presented as mean + SEM. Two-way ANOVA, Tukey's post hoc test. **** $p \leq 0.0001$.

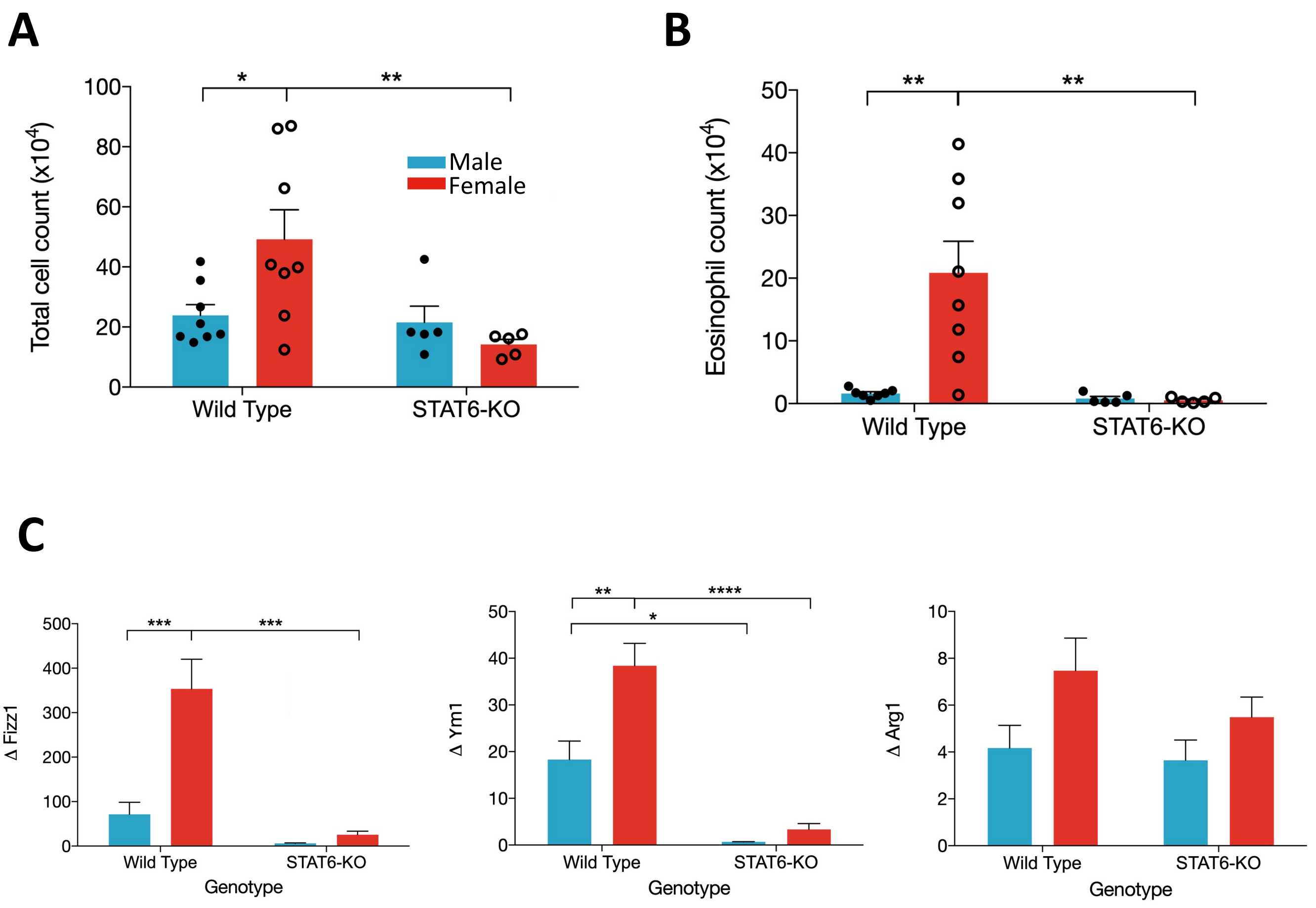

Supplemental Fig 1

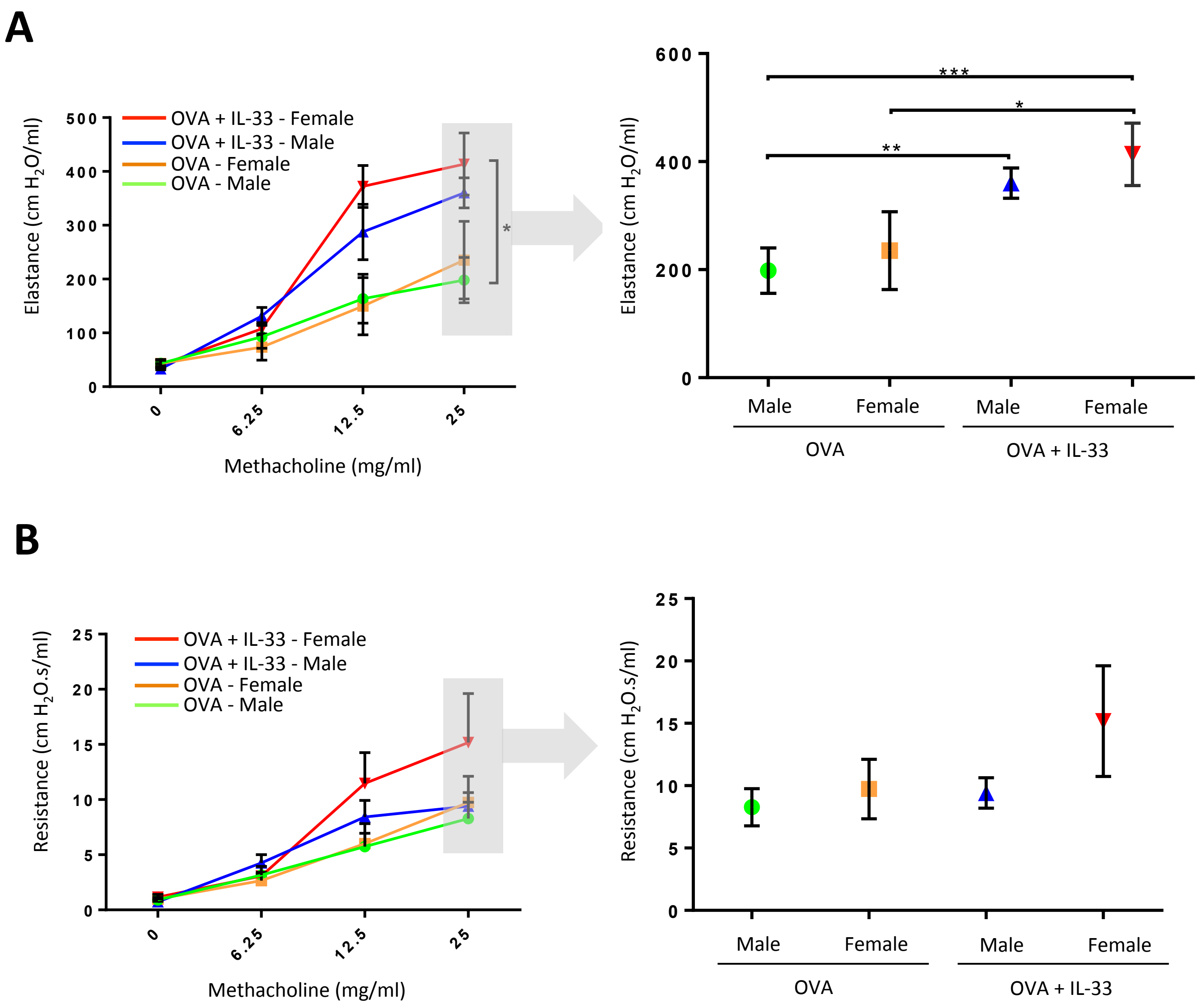

Supplemental Fig 2

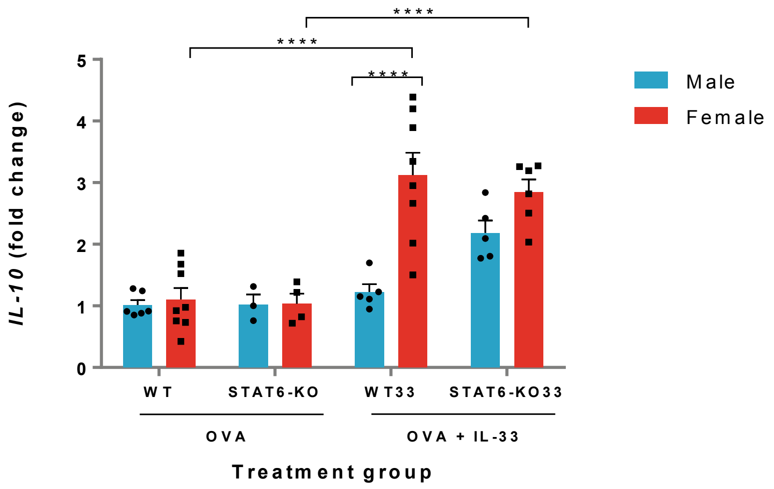

Supplemental Fig 3
